# Allele Identification for Transcriptome-Based Population Genomics in the Invasive Plant *Centaurea solstitialis*

**DOI:** 10.1534/g3.112.003871

**Published:** 2013-02-01

**Authors:** Katrina M. Dlugosch, Zhao Lai, Aurélie Bonin, José Hierro, Loren H. Rieseberg

**Affiliations:** * Department of Botany, University of British Columbia, Vancouver, BC V6T1Z4 Canada; † Department of Ecology and Evolutionary Biology, University of Arizona, Tucson, Arizona 85721; ‡ Department of Biology and Center for Genomics and Bioinformatics, Indiana University, Bloomington, Indiana 47405; § Facultad de Ciencias Exactas y Naturales, INCITAP (CONICET-Universidad Nacional de La Pampa), AR-6300, Santa Rosa, La Pampa, Argentina

**Keywords:** normalized ESTs, allele clustering, 454 GS FLX Titanium, yellow starthistle, invasive species

## Abstract

Transcriptome sequences are becoming more broadly available for multiple individuals of the same species, providing opportunities to derive population genomic information from these datasets. Using the 454 Life Science Genome Sequencer FLX and FLX-Titanium next-generation platforms, we generated 11−430 Mbp of sequence for normalized cDNA for 40 wild genotypes of the invasive plant *Centaurea solstitialis*, yellow starthistle, from across its worldwide distribution. We examined the impact of sequencing effort on transcriptome recovery and overlap among individuals. To do this, we developed two novel publicly available software pipelines: SnoWhite for read cleaning before assembly, and AllelePipe for clustering of loci and allele identification in assembled datasets with or without a reference genome. AllelePipe is designed specifically for cases in which read depth information is not appropriate or available to assist with disentangling closely related paralogs from allelic variation, as in transcriptome or previously assembled libraries. We find that modest applications of sequencing effort recover most of the novel sequences present in the transcriptome of this species, including single-copy loci and a representative distribution of functional groups. In contrast, the coverage of variable sites, observation of heterozygosity, and overlap among different libraries are all highly dependent on sequencing effort. Nevertheless, the information gained from overlapping regions was informative regarding coarse population structure and variation across our small number of population samples, providing the first genetic evidence in support of hypothesized invasion scenarios.

Almost five decades of molecular genetic research has revealed that an abundance of genetic variation resides in the natural populations of most living organisms (*e.g.*, Lewontin and Hubby 1966; Hamrick and Godt 1989; Morin *et al.* 2004; Ossowski *et al.* 2008; Durbin *et al.* 2010). This variation is necessarily shaped by the history of mating, demography, dispersal, and adaptation playing out within species and as a result allele frequency distributions in wild populations can provide unique insights into evolution and ecology (Avise 2004; Wakeley 2008). Most recently, genome-wide studies of allelic variation in natural populations are proving to be particularly powerful for detecting subtle and/or complex aspects of population structure, selection on specific regions of the genome, and associations between allelic and phenotypic variation (*e.g.*, Drosophila 12 Genomes Consortium 2007; McCarthy *et al.* 2008; Zayed and Whitfield 2008; Emerson *et al.* 2010; Hancock *et al.* 2010). Genomic approaches were first accessible to population geneticists via targeted amplification of genome-wide markers (Vos *et al.* 1995), but the declining cost and increasing length of next-generation sequencing reads are now making bulk sequencing of the genome practical for allele discovery in nonmodel and outbred study subjects (Li *et al.* 2008; Davey *et al.* 2011; Nielsen *et al.* 2011).

Sequencing of transcribed genes, the ‘transcriptome,’ is an especially attractive strategy for surveying genomic variation at a relatively low cost because it provides a reduced fraction of the genome that is also rich in information (Bouck and Vision 2007). Coding sequences can be placed into reading frame, aspects of protein variation studied, and their general function inferred from homology to proteins in model organisms (Wheat 2010). Coding regions also may underlie phenotypic variation of interest and the associated protein variants identified as the direct targets of selection (*e.g.*, Elmer *et al.* 2010; Renaut *et al.* 2010; Barreto *et al.* 2011). Simultaneously, a genome-wide panel of transcriptome-derived SNPs should also be largely suitable for standard population genetic analyses that assume neutrality (*e.g.*, Barbazuk *et al.* 2007; Seeb *et al.* 2011; Ueno *et al.* 2010; Zakas *et al.* 2012).

Here, we explore the potential for *de novo* whole transcriptome sequences from many individuals to generate useful genetic polymorphism data across their genomes. We focus on a species of economic and ecological concern, yellow starthistle (*Centaurea solstitialis* L., Asteraceae). Yellow starthistle (hereafter YST) is an annual herb, native to Eastern Europe and the Caucasus, and hypothesized to be naturalized in Mediterranean Western Europe (Maddox *et al.* 1985). Historical records indicate that this species was introduced to South America as a crop contaminant via Spain in the mid-1600s and then to North America (primarily via Chile) in the 1800s (Gerlach 1997). Introduced YST have successfully invaded a wide variety of grass- and shrublands in western North America and temperate South America. In North America, this species has spread to 23 U.S. states and five Canadian provinces (Maddox *et al.* 1985), with the largest infestations occurring in California (>15 million acres) and the Pacific Northwest (>3.3 million acres) United States (Wilson *et al.* 2003).

For transcriptome sequencing, we generated normalized cDNA libraries from actively growing tissues in an effort to sequence as many of the coding regions of the genome as possible. In contrast to direct sequencing of cDNA for quantification of gene expression (*i.e.*, ‘RNASeq’), normalization reduces the representation of common transcripts for better sequence coverage of both common and rare transcripts (Zhulidov *et al.* 2004; Christodoulou *et al.* 2011). We report on sequences of 40 YST plants: 19 from its invaded range (11 from North America and 8 from South America), 4 from its putative ancient naturalized range (Spain), and 17 from its native range (Eastern Europe/Caucasus). Plants are diploid (2N = 16) throughout these regions (Heiser and Whitaker 1948; Widmer *et al.* 2007; Ozturk *et al.* 2009), with a genome size of 1N = 851 Mbp (Brancheva and Greilhuber 2006).

We address two key issues using our dataset: (1) the accurate identification of allelic variation in these outbred individuals, and (2) the extent of sequence overlap among transcriptome libraries. YST propagates exclusively by seed and is self-incompatible (Sun and Ritland 1998), and so we expect allelic variation and observed heterozygosity to be relatively high in our populations (Sun 1997). Accurate identification of allelic variants of the same locus is not straightforward, however, because allele sequences vary widely in their divergence (*e.g.*, Lawlor *et al.* 1988; Li and Sadler 1991; Moriyama and Powell 1996; Bergelson *et al.* 2001), overlapping the divergence of closely related but separate loci (paralogs) (Lynch and Conery 2000; Sebat *et al.* 2004; Hahn *et al.* 2005; Demuth *et al.* 2006; Hahn *et al.* 2007). This means that it is impossible to discriminate among alleles and paralogs by sequence divergence alone. We present a conservative strategy to filter for unique loci by using the putative allelic variation across all individuals under study to identify the minimum number of haplotypes within individuals. This strategy relies on phasing of multi-locus SNPs within individual genes, and so we use a long-read 454 pyrosequencing approach to increase our ability to discriminate accurately among haplotypes. We find that approximately one-half of our putative loci are suitable for population genetic analyses, that allele recovery is far more dependent on sequencing depth than is gene recovery, but that modest transcriptome sampling nevertheless generates thousands of informative markers observed across many individuals. In our study system, these markers indicate a history of admixture and the presence of high genetic variation in introduced populations of YST.

## Materials and Methods

### Library preparation

We prepared cDNA libraries from leaves or whole plants of 8-wk-old seedlings (n = 40), representing the native and introduced range of YST (Table 1). Seeds from these populations were reared in a greenhouse at 25° and 16-hr day/8-hr night conditions in a 1:1 mixture of sand and potting soil. RNA extraction followed Lai *et al.* (2012).

**Table 1:** Sequencing effort and assembly information for *C. solstitialis* normalized Roche 454 transcriptome libraries

						Raw	Cleaned			Assembled				
Sample^Tissue^ [Latitude, Longitude]	Platform	Plates	Mb	Mb	Read No.	Median bp	Mb	Unigene No.	Median bp	Contig No.	UCO No.
North America (introduced)											
CA-1-1^S^ [N 41° 59’, W 122° 36’]	FLX	0.25	17.2	13.0	69939	211	2.4	9783	242	8769	10
CA-1-2^S^ [N 41° 59’, W 122° 36’]	FLX	0.25	25.1	19.0	101468	213	3.2	12810	242	11237	18
CA-2-2^S^ [N 40° 25’, W 122° 16’]	FLX	0.25	20.1	15.9	82739	216	2.8	10596	255	9236	25
CA-2-4^S^ [N 40° 25’, W 122° 16’]	FLX	0.25	19.9	14.3	78770	210	2.4	9853	238	8551	14
CA-3-1^S^ [N 39° 12’, W 121° 06’]	FLX	0.5	58.0	45.6	229635	224	7.1	23671	268	19905	77
CA-3-2^S^ [N 39° 12’, W 121° 06’]	FLX	0.5	58.1	39.9	212773	219	5.8	21578	254	18377	28
CA-4-1^S^ [N 38° 31’, W 121° 45’]	FLX	0.5	80.1	66.8	321955	226	10.1	32206	270	27121	109
CA-4-3^S^ [N 38° 31’, W 121° 45’]	FLX	0.25	20.0	13.2	72154	214	2.3	9524	238	8655	11
CA-4-4^S^ [N 38° 31’, W 121° 45’]	Ti	0.75	206.3	193.2	579859	366	24.8	42042	537	32786	252
CA-5-3^S^ [N 38° 16’, W 121° 49’]	FLX	0.5	80.2	65.5	325569	223	9.9	32743	266	27222	111
CA-5-4^S^ [N 38° 16’, W 121° 49’]	FLX	0.25	21.1	14.7	79481	215	2.3	9172	249	8112	13
South America (introduced)											
AR-1-24^L^ [S 36° 26’, W 64° 17’]	Ti	0.5	234.7	225.4	542881	462	33.4	46538	632	38346	299
AR-1-25C^L^ [S 36° 26’, W 64° 17’]	Ti	0.56	153.7	132	369437	412	21.1	39579	532	29944	177
AR-6-13^L^ [S 37° 39’, W 64° 08’]	Ti	0.5	256.4	247.1	596060	473	28.5	38572	630	31082	287
AR-6-26^L^ [S 37° 39’, W 64° 08’]	Ti	0.5	181.7	169.2	485202	390	17.3	30641	537	23376	185
AR-8-15^L^ [S 38° 11’, W 64° 04’]	Ti	0.5	166.1	154.6	443044	391	20.4	35628	549	26419	230
AR-8-19^L^ [S 38° 11’, W 64° 04’]	Ti	0.5	259.3	249.9	600878	458	28.7	37199	673	30305	303
AR-13-24^L^ [S 36° 18’, W 65° 40’]	Ti	0.5	274.6	264.7	622982	491	28.4	37408	679	29348	311
AR-13-28^L^ [S 36° 18’, W 65° 40’]	Ti	0.63	183.5	171.5	488746	405	21.1	38160	540	28579	206
Western Europe (putative ancient expansion)											
SP-1-5^L^ [N 39° 50’, W 2° 30’]	Ti	0.5	152.5	135.6	393803	398	19.5	38699	519	27841	172
SP-1-10^L^ [N 39° 50’, W 2° 30’]	Ti	0.5	269.7	258.7	604697	483	29.5	43236	637	32884	292
SP-2-2^L^ [N 41° 45’, W 4° 5′]	Ti	0.63	181.9	162.1	522160	341	22.8	46071	474	34707	184
SP-2-6^L^ [N 41° 45’, W 4° 5′]	Ti	1	332.6	315.9	815538	459	41.6	62444	583	48230	311
Eastern Europe (native)											
GA-1-1^S^ [N 41° 56’, E 45° 27’]	FLX	0.25	19.1	12.5	71584	209	2.0	8901	233	7823	8
GA-2-1^S^ [N 41° 56’, E 45° 35’]	FLX	0.25	18.9	12.9	73656	210	2.0	8921	233	7659	11
GA-3-4^S^ [N 41° 44’, E 45° 12’]	FLX	0.25	11.5	7.5	42595	212	1.3	5257	240	4704	3
GA-4-3^S^ [N 41° 43’, E 45° 16’]	FLX	0.25	13.0	7.7	43567	215	1.3	5299	240	4811	11
GA-5-4^S^ [N 41° 38’, E 45° 38’]	FLX	0.25	12.8	8.6	48417	214	1.5	6073	243	5407	4
GA-5-24^L^ [N 41° 38’, E 45° 38’]	Ti	0.63	153.5	126.5	367110	390	17.4	33086	522	25382	165
HU-1-8^L^ [N 46° 58’, E 18° 41’]	Ti	0.5	185.3	172.4	465081	421	22.3	39661	559	28967	213
HU-2-10^L^ [N 47° 19’, E 21° 02’]	Ti	0.5	297.9	286.6	661490	489	32.5	40448	699	32643	311
RO-1-6^L^ [N 47° 42’, E 26° 2’]	Ti	0.75	189.6	170.8	518480	376	22.6	43834	510	32820	182
RO-5-10^L^ [N 46° 15’, E 27° 39’]	Ti	0.5	292.5	279.1	663787	479	33.7	52108	602	39456	293
TK-1-3^S^ [N 39° 45’, E 29° 06’]	FLX	0.5	24.7	17.3	89230	229	3.2	11053	268	9841	26
TK-1-5^L^ [N 39° 45’, E 29° 06’]	Ti	0.63	159.5	145.6	494855	327	20.2	41229	462	30361	207
TK-2-3^S^ [N 37° 02’, E 29° 47’]	FLX	0.25	17.1	10.6	63668	201	1.8	8085	223	7106	4
TK-2-4^S^ [N 37° 02’, E 29° 47’]	FLX	0.25	15.7	10.0	59734	204	1.5	7087	225	6191	6
TK-3-2^S^ [N 37° 50’, E 27° 51’]	FLX	0.25	11.4	6.2	38205	198	1.0	4873	210	4373	2
TK-5-4^S^ [N 37° 01’, E 30° 22’]	Ti/FLX	2.25	430.7	389.4	1258319	352	35.1	71045	418	48608	278
TK-5-9^L^ [N 37° 01’, E 30° 22’]	Ti	0.5	252.1	241.7	554227	481	29.8	40147	671	32773	283
Pseudo-reference							39.6	43717	811		260
Pseudo-reference filtered for polymorphic single loci							21.2	22687	840		154

Tissues included are whole seedlings (S) or leaves (L). FLX, 454 Life Science Genome Sequencer FLX; Ti, FLX-Titanium.

To generate the full-length complementary DNA (cDNA) for the transcriptome analysis, we used a protocol from the Clontech Creator SMART cDNA library construction kit (Clontech Laboratories, Mountain View, CA). This requires an oligo-dT primer that anchors the polyA tail of mRNA to primer the cDNA synthesis process. However, mononucleotide runs reduce sequence quality and quantity due to excessive light production and crosstalk between neighboring cells (Margulies *et al.* 2005). To counteract this problem, we used two different approaches to synthesize cDNA. The first approach was to use a “broken chain” short oligo-dT primer (primer sequence: 5′- AAGCAGTGGTATCAACGCAGAGTCGCAGTCGGTACTTTTTTCTTTTTTV-3′, V = A, G, or C) to prime the poly(A) tail of mRNA during first-strand cDNA synthesis (Meyer *et al.* 2009). In the second approach we used two different modified oligo-dT primers: one (5′-AAGCAGTGGTATCAACGCAGAGT(T)_4_G(T)_9_C(T)_10_VN-3′) to prime the poly(A) tail of mRNA during first strand cDNA synthesis and another (5′-AAGCAGTGGTATCAACGCAGAGT(T)_4_GTC(T)_4_GTTCTG(T)_3_C(T)_4_VN-3′) to further break down the stretches of poly(A) sequence during second strand cDNA synthesis (Beldade *et al.* 2006). Approximately 1.5 µg of total RNA was reverse-transcribed to first-strand cDNA using these methods.

Double-stranded (ds) cDNA synthesis was performed using Phusion polymerase (New England Biolabs, Ipswich, MA) with a hot start of 98° for 30 sec, followed by 18 cycles of 98° for 7 sec, 66° for 20 sec, and 72° for 4 min. The ds-cDNA polymerase chain reaction product was purified using a QIAquick PCR Purification column (QIAGEN). Normalization was performed using a TRIMMER-DIRECT cDNA normalization kit (Evrogen, Moscow, Russia). Approximately 600−1200 ng of purified ds-cDNA was used as the starting amount for normalization. A mixture of 0.25 µL and 0.5 µL of DSN normalization tubes was used for the first and second amplifications.

After normalization, cDNA was fragmented to 500- to 800-bp fragments by sonication or nebulization and size-selected to remove small fragments using AMPure SPRI beads. The fragmented ends were polished and ligated with adaptors (Meyer *et al.* 2009). The optimal ligation products were selectively amplified and subjected to two rounds of size selection including gel electrophoresis and AMPure SPRI bead purification (Lai *et al.* 2012).

### Sequencing and assembly

All cDNA libraries were sequenced on the 454 Life Science Genome Sequencer (Roche Applied Science, Branford, CT), using either FLX or FLX-Titanium chemistry (Table 1). Sequencing of each sample was performed by the 454 Life Science Sequencing Center at Roche Applied Science, the Center for Genomics and Bioinformatics at Indiana University, or the Genome Quebec Innovation Centre at McGill University. Primer/adapter and polyA/T sequences were trimmed from the reads using custom Perl trimming scripts and SeqClean (http://www.tigr.org/tdb/tgi/software/). Sequences composed of primer multimers were removed using TagDust (Lassmann *et al.* 2009), with a false discovery rate of 0.01. These cleaning steps were combined into a flexible automatic sequence cleaning pipeline ‘SnoWhite’ (v1.1.4), which we have made publicly available at http://evopipes.net (Barker *et al.* 2010).

Cleaned reads were assembled into contigs *de novo* using the assembler MIRA v3.0 (Chevreux *et al.* 2004). MIRA produced identical duplicate contigs in areas with high read depth, and these were merged using additional iterations of both MIRA and CAP3 (Huang and Madan 1999) at 97% minimum similarity, using the pipeline iAssembler v1.3 (Zheng *et al.* 2011). Successful resolution of highly similar alleles and/or paralogs into unique contigs was verified by examining synonymous site divergence among gene family members using the program DupPipe (Barker *et al.* 2008, 2010). Resolution of highly similar sequences within eukaryotic individuals should yield gene family phylogenetic trees with a characteristic L-shaped distribution of many recent nodes and diminishing numbers of older nodes (Lynch and Conery 2000).

### Allele clustering with AllelePipe

**Figure 1:**
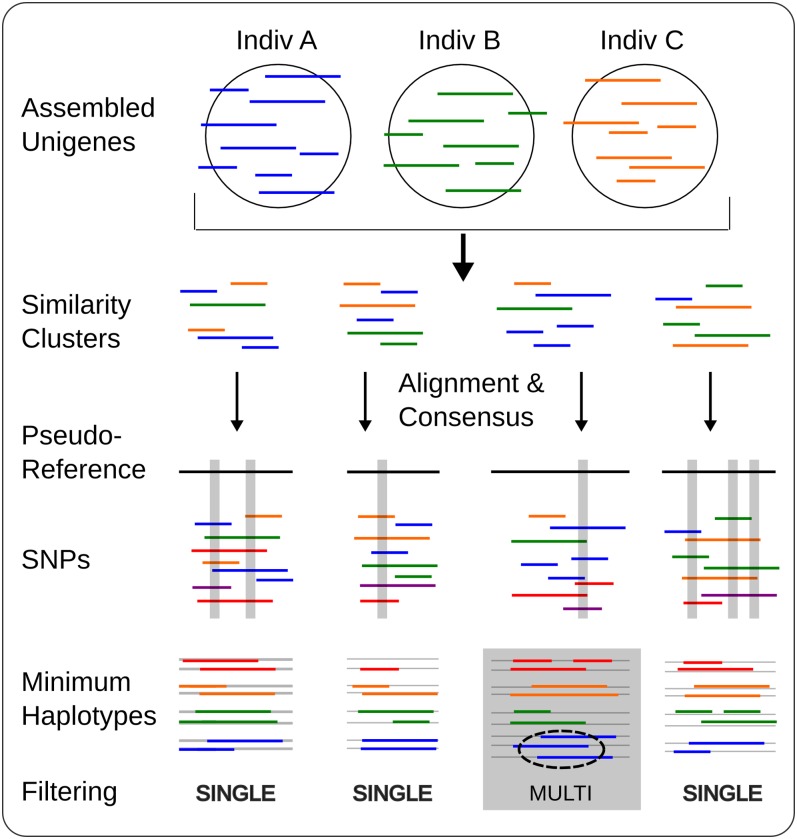
AllelePipe workflow for identifying alleles without a reference genome. Unigenes from all individuals are pooled and clustered by similarity. Clustered sequences are aligned and consensus sequences are generated, providing a pseudo-reference genome. Unigenes from the same and/or different individuals are aligned to the reference, and SNPs are identified. Multilocus SNP information is used to construct a minimum set of haplotypes for each individual, and clusters in which individuals are represented by an excess number of putative alleles are flagged as potential multigene clusters.

Allele and single-nucleotide polymorphism (SNP) identification typically rely on mapping reads to a reference genome that represents a haploid set of genes (Bentley 2006; Charlesworth 2010). YST lacks a reference genome, and allelic variation in our samples prevented simple *de novo* creation of an accurate haploid reference sequence library. To circumvent this problem, we developed a novel software pipeline to cluster putative alleles within and among individuals, available as ‘AllelePipe’ (v1.0.25; Figure 1) at http://evopipes.net. Using the AllelePipe, we assessed similarity among all sequences from all 40 individuals with SSAHA2 (Ning *et al.* 2001), with 95% minimum similarity and 300-bp minimum alignment length between sequences. We also included sequences from a published assembly of a Sanger EST library for a YST genotype from the invasion in central California (Genbank #EH750647-EH791053) (Barker *et al.* 2008). AllelePipe was used to verify that similar sequences aligned throughout their region of overlap (expected for true alleles), and to cluster groups of similar sequences via single-linkage clustering. Single-linkage clustering generates maximal aggregation of sequences, and will bring together both closely related paralogs and their alleles (Dlugosch and Bonin 2012). Multiple alignments were created for sequences within each cluster and their consensus sequence generated using CAP3. Clusters were discarded from the analysis if they aggregated increasingly dissimilar sequences, preventing a single CAP3 consensus. From the resulting consensus sequences, a genomic ‘pseudo-reference’ FASTA file was generated for the entire dataset, suitable for anchoring contig alignments across individuals. We evaluated the quality of our overall clustering strategy by aligning our consensus sequences with known highly conserved eukaryotic single copy loci, including 357 ultra-conserved orthologs (UCOs, available at http://compgenomics.ucdavis.edu/compositae_reference.php; Kozik *et al.* 2008), using tblastx comparisons with maximum expectation (e-value) of 0.1 and minimum 30 protein residue alignments (Blast v2.2.24) (Altschul *et al.* 1997). Only one-to-one matches between UCOs and clusters are expected for properly clustered loci.

Finally, we filtered for (putatively) valid loci by removing those clusters with evidence of more than two alleles (multi-SNP haplotypes across the entire gene region) from any individual in the dataset. Excess alleles suggest that the cluster is not a single locus and instead a group of paralogous loci. This approach leverages the information gained from multiple individuals to infer paralogy, where it is not possible to do so from genomic DNA sequencing depth or alignment to a complete reference genome. Again using the AllelePipe, we identified SNPs for each sequence against the pseudo-reference by using ssahaSNP (Ning *et al.* 2001) with minimum 90% similarity for alignment to the pseudo-reference. Singleton SNPs (those seen only once across the dataset) were removed as potential errors, and the number of unique haplotypes for each individual in the cluster were evaluated for evidence of paralogous clustering. Those clusters with no more than two haplotypes per individual were retained, and their SNP variation (from ssahaSNP) retained for further analyses.

### Gene annotation

Gene Ontology (GO) classifications for our pseudo-reference sequences and individual transcriptome assemblies were obtained through BLASTx searches against the *Arabidopsis thaliana* protein database from The Arabidopsis Information Resource (release TAIR10_pep_20101214; http://www.arabidopsis.org/), using an e-value cut off 1*10^−10^. To evaluate the impact of sequencing effort on the gene content of assemblies, we compared the representation of GO categories across libraries using a Chi-squared Contingency test in R (R Development Core Team 2010).

### Population genetic analyses

We examined the impact of both sequencing effort and source region (native *vs.* invading) on assembled contig numbers, observed SNP positions, observed heterozygosity, and SNP overlap. SNP frequencies and overlap among individuals were quantified using custom Perl scripts. Significance of relationships was tested with linear model fits in R, using the lm function (R Development Core Team 2010). Geographic partitioning of genetic variation in the native range was assessed with STRUCTURE v2.3.2.1 for 1-4 subpopulations (K), using 10,000 burnin steps and 10,000 iterations of the models (Pritchard *et al.* 2000; Falush *et al.* 2003). Runs were repeated three to five times at each K to verify convergence. Naturalized and invading samples were then assigned to native sub-populations using STRUCTURE, and results were plotted using Distruct v 1.1 (Rosenberg 2004).

## Results and Discussion

### Transcriptome recovery

**Figure 2:**
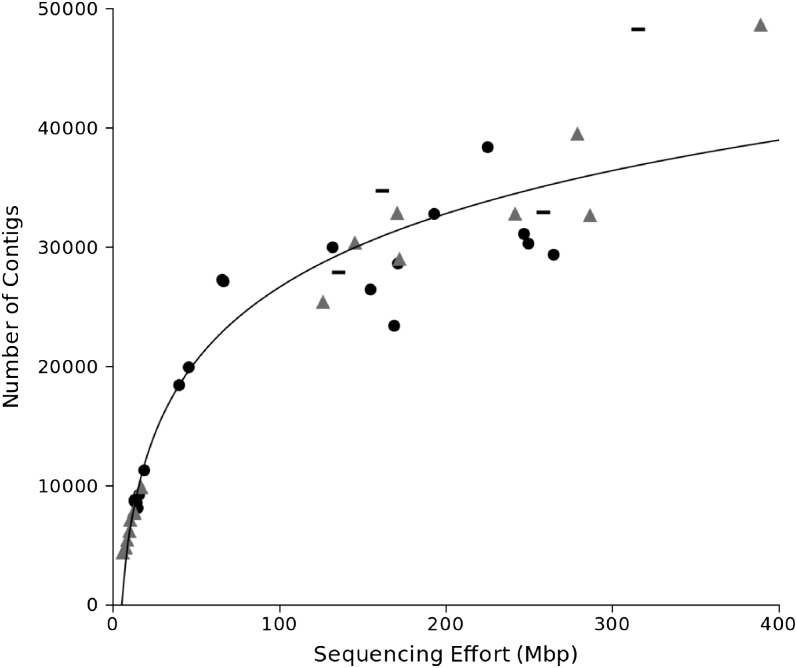
Contig numbers in transcriptome libraries of native (triangles), naturalized (dashes), and invading (circles) genotypes, as a function of total sequence effort after cleaning by SnoWhite. A logarithmic fit is shown to variation across all individuals.

We obtained between 11.4 and 430.7 Mbp of raw sequence per individual as a result of a range of sequencing efforts across our samples (Table 1; NCBI Short Read Archive accession #SRA059334; assembly for AR-13-24 previously published in Lai *et al.* 2012 and available at doi:10.5061/dryad.cm7td/4). Our assemblies generated up to 71,054 unigenes (contigs and singleton reads combined), including up to 48,230 contigs (Figure 2). Gene number in the YST genome is not yet known, but our largest assemblies are at the top of the range of current annotation counts among complete angiosperm genomes (Barker *et al.* 2012), and somewhat higher than the ~35,000 loci predicted in the genome of the related asterid tomato (The Tomato Genome Consortium 2012). Given that our accessions are outbred individuals and allelic variation is expected, these numbers are consistent with a relatively complete view of expressed genes in this species. Indeed we find patterns of saturating gains in unique sequences and coverage of known conserved loci: Both unigene and contig numbers were positively related to sequencing effort, and these relationships were fit closely by logarithmic curves (regressions: unigenes, R^2^ = 0.93, *P* < 0.0001; contigs, R^2^ = 0.91, *P* < 0.0001, Figure 2), indicating that recovery of additional unique sequences was associated with exponential increases in sequencing effort. Although the longer-read GSFLX-Titanium chemistry resulted in longer contigs (Table 1), our contig numbers generated from those libraries followed the same logarithmic relationship with sequencing effort predicted by the shorter-read GS-FLX chemistry (nonsignificant interaction of chemistry type and sequencing effort in linear model: *P* = 0.43; Supporting Information, Figure S1). The proportion of UCOs recovered reached 87% (311 loci) among the largest libraries (Table 1). In general, gains from additional sequencing were relatively modest above approximately 100 Mb of usable sequence, which is likely to be less than 5x coverage of the YST transcriptome, given our assembly size. This result suggests that representative information can be gained from low coverage datasets, even while additional sequencing depth continues to yield further gene discovery (Lai *et al.* 2012).

To evaluate our ability to resolve highly similar sequences in our assemblies, we examined frequency distributions of the synonymous site divergence at nodes in gene family trees for each of our datasets. Distributions of divergence times showed expected L-shaped patterns of abundant recent ancestry in each case, consistent with successful resolution of closely-related paralogs and alleles in our assemblies (Figure S2). The distributions also accurately reveal an additional small peak at ~Ks = 0.65, which has been shown previously to correspond to an ancient genome duplication event near the base of the family, based upon Sanger sequence data (Barker *et al.* 2008). Consistent with those previous analyses, there was no evidence of more recent genome duplication events in our YST individuals.

### Allele clustering and library overlap

Single-linkage clustering of unigenes across all individuals using AlellePipe generated 43,717 total putative loci with a median consensus length of 811 bp. These aligned to 260 (73%) of the UCOs, and the majority of these UCOs aligned with only one cluster as expected (Figure S3). Alignment of the remaining UCOs to multiple clusters could reflect sequence divergence (low similarity with UCOs), duplication of the locus, or failure of the reads to cluster together. Divergence or duplication is possible but relatively unlikely in such highly conserved “single-copy” loci (Duarte *et al.* 2010); it is more likely that splitting of these clusters resulted from alternate splicing (Barbazuk *et al.* 2008) or insufficient overlap for observing similarity among sets of sequences. These latter processes will introduce clusters of sequences that are incomplete and/or redundant but nevertheless accurate variations on a gene region or splice form. Gene annotation of the consensus cluster sequences by similarity to *A. thaliana* proteins yielded 26,728 matches, 11,268 of which (25.8% of all clusters) were identified as unique, nonredundant annotations. Similar rates of homology to *A. thaliana* were observed in other transcriptome surveys of asterid plants, using both Sanger and next-generation sequencing platforms (Barker *et al.* 2008; Dempewolf *et al.* 2010; Angeloni *et al.* 2011; Lai *et al.* 2012).

After clustering, AllelePipe identified 22,687 polymorphic unigenes that conformed to expectations of no more than two alleles per individual. These loci annotated to 23,896 *A. thaliana* genes, indicating that most of the inferred multilocus clusters were not those that had successfully annotated, perhaps due to poor consensus formation. For annotated loci, GO representation differed among inferred single loci and all clusters (*P* < 0.001 for all three categories; Figure S4). Interestingly, single loci included a lower representation of transcriptome factors, and a greater representation of intracellular and plastid-associated components. These patterns contrast with patterns of duplicate retention from paleo genome duplication events in the Compositae family, consistent with the hypothesis that certain complete pathways are retained in duplicate from whole genome duplications due to dosage constraints, while other functions are free to vary in copy number [and the latter would generate our multilocus clusters (Barker *et al.* 2008, 2012) and references therein].

**Figure 3:**
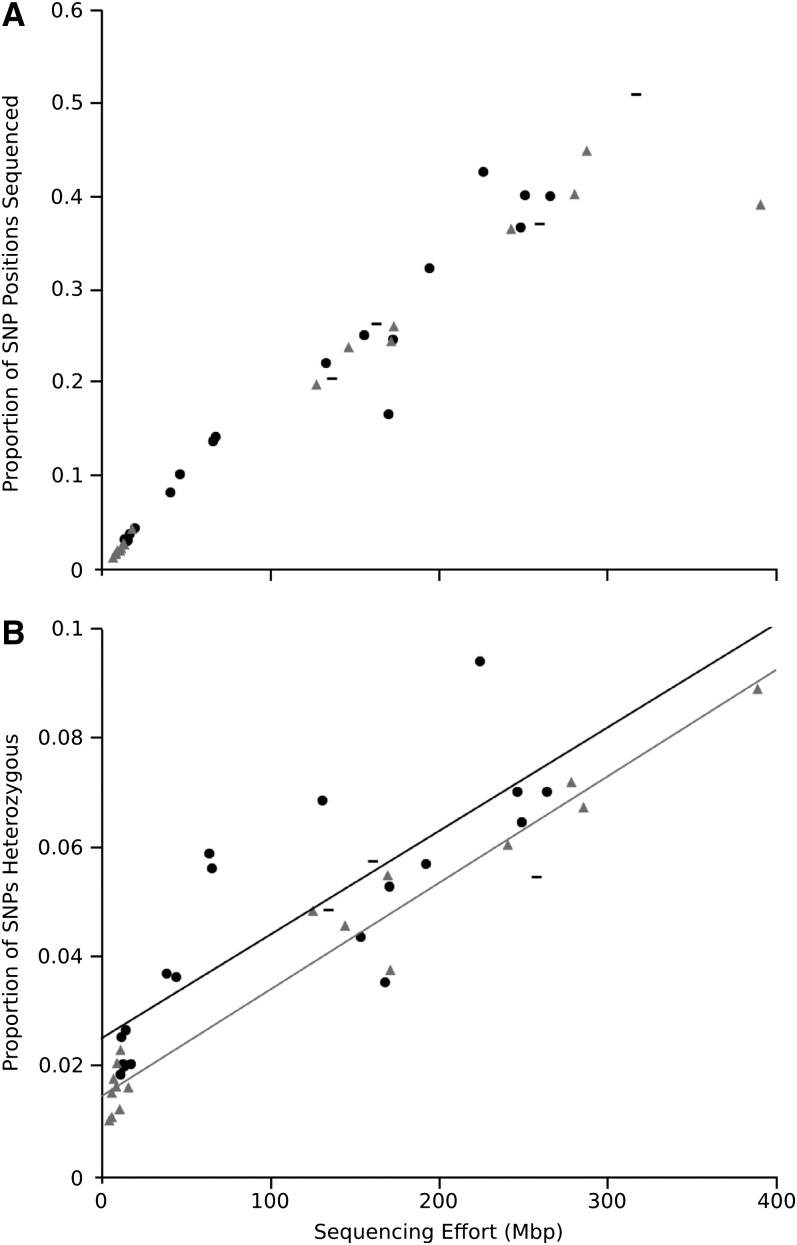
Coverage of SNP positions identified across the dataset. (A) Proportion of SNPs that were sequenced in each individual, and (B) frequency of observed heterozygous loci among sequenced SNPs within native (triangles), naturalized (dashes), and invading (circles) individuals, relative to total sequencing effort after read cleaning. Linear fits are shown to native (gray line) and invading (black line) individuals.

Aligning sequences from the 41 YST datasets against this filtered set of 22,687 pseudo-reference sequences revealed 237,034 polymorphic sites over the total sequence length of 21.2 Mbp, where each SNP variant was observed at least twice across all haplotypes (1 SNP per 89.6 bp across the sample). This low SNP density underscores the need for long-read approaches to accurately recover haplotypes within and among conspecific individuals (Dlugosch and Bonin 2012; Lai *et al.* 2012). The proportion of these sites sequenced in each individual increased sharply with sequencing effort (Figure 3A), with no indication of saturation; the largest libraries covered less than 60% of SNP positions. The number of observed heterozygous positions also showed a strong and linear increase with sequencing effort in both native and invading samples (Figure 3B). The accurate observation of heterozygosity is likely to be particularly important both for revealing population genetic information, and for validating the SNPs themselves (Seeb *et al.* 2011). Thus, despite saturating returns of additional unique sequences in the larger libraries, our ability to observe allelic variation at these loci did not plateau, and analyses of this type of variation must take sequencing effort into account.

**Figure 4:**
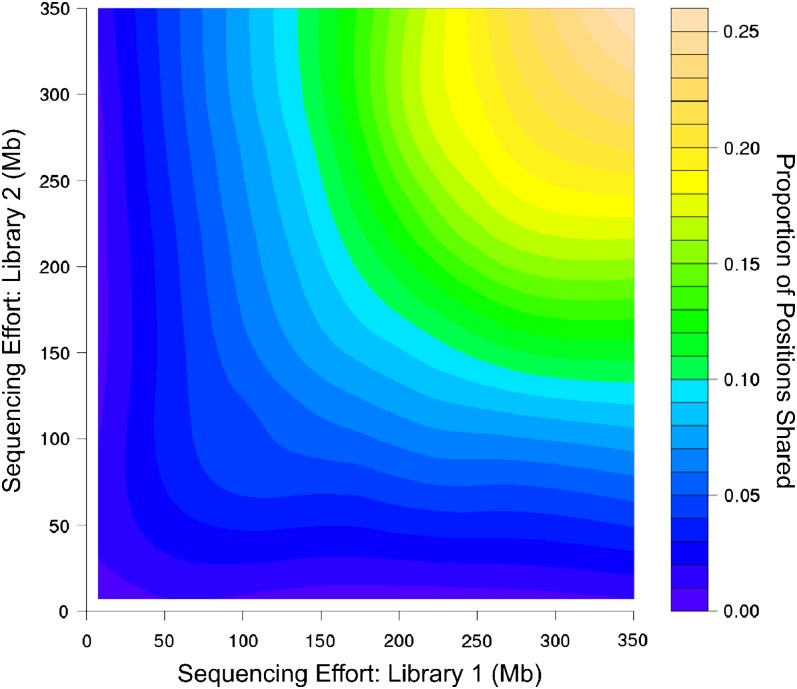
Pairwise overlap in observed SNPs. Isoclines reflect the proportion of all SNP positions that are observed in pairwise comparisons of 40 transcriptome libraries, as a function of sequencing effort in both samples.

**Figure 5:**
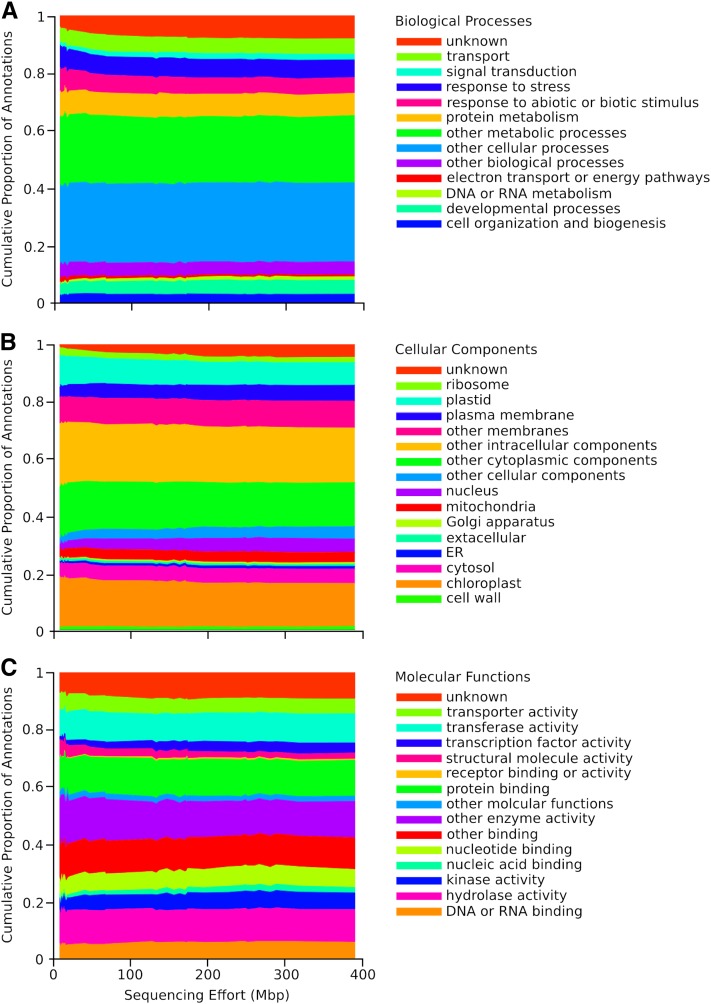
The distribution of GO annotations to *A. thaliana* as a function of transcriptome sequencing effort.

Sequence overlap among samples also was improved by increased sequencing effort. In pairwise comparisons, the proportion of all polymorphic positions shared between individuals increased from <1% to almost 30% in the largest libraries (which shared nearly 70% of the SNP positions observed in any individual library; Figure 4). The 10 largest libraries included four native, four invading, and two naturalized individuals with more than 200 Mbp of sequencing effort each. Surveys of SNP sites—as identified from across the dataset—within just these greatest coverage libraries revealed that most SNP sites were sequenced in only a few individuals, though 6883 loci were present in all 10 libraries (Figure S5). The number of overlapping SNP positions was significantly greater than expected by chance, given the number of positions observed in each library (K-S Goodness of Fit test: *P* < 0.01, Figure S5). Overlapping positions are almost certainly biased toward the most commonly expressed loci, however our GO categorizations indicate that this does not represent a particularly biased subset of genome function. Functional categorization and distribution of the annotations within the three GO categories (biological process, cellular component, and molecular function), did not vary more than a few percent among the individual assemblies for any classification, though this modest variation was statistically significant (*P* < 0.0001 among libraries within each of the three categories, Figure 5). There was a consistent trend toward greater representation of loci of unknown function (although still similar to *A. thaliana* proteins) in larger libraries within each category (Figure 5), indicating greater recovery of less well-studied and presumably more rarely-expressed loci.

### Novel insights into YST population genetics

For introduced and invasive species, identifying the sources of their genetic variation can provide important insights into the circumstances that made these introductions so successful (Dlugosch and Parker 2008; Prentis *et al.* 2008; Lai *et al.* 2012). Native individuals assembled slightly higher numbers of both unigenes and contigs (Figure 2) than invading individuals, potentially indicating reduced sequence variation in introduced genotypes, but these differences were not significant in either case (analysis of covariance on Ln-transformed data: unigenes, *P* = 0.06; contigs, *P* = 0.23). Instead, the identification of allelic variation among our samples suggested different population genetic inferences in our YST transcriptome dataset: Across all inferred SNP positions, invaders had significantly greater heterozygosity than natives (analysis of covariance : F_2,33_ = 82.21, *P* = 0.005, Figure 4B) and a greater variance around predicted values based on sequencing effort (linear regressions: invader R^2^ = 0.67, native R^2^ = 0.96). These patterns are consistent with a known history of multiple, large introductions of YST (Gerlach 1997), which should have established substantial genetic diversity in the invaded range.

**Figure 6:**
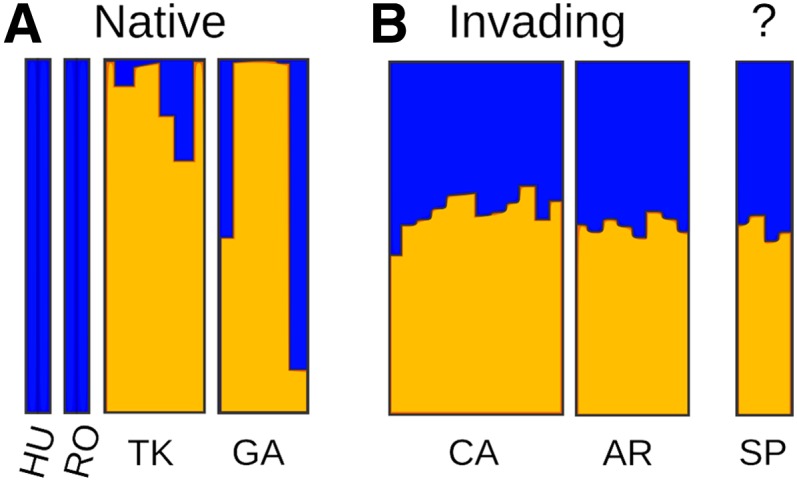
STRUCTURE populations inferred from SNP variation. Vertical bars show the population assignment (color) for each individual by region. (A) Two major genetic groups (blue and orange) are supported for the native range, based upon genotypes from Hungary (HU), Romania (RO), Turkey (TK) and the Republic of Georgia (GA). (B) Admixture of these sources is suggested for both putatively naturalized genotypes in Spain (SP) and invading genotypes from California (CA) and Argentina (AR), when the two native genetic groups are fixed as potential source populations. SNP frequencies were based upon 2568 positions observed in at least five individuals per continent.

With the use of 2568 SNPs that were genotyped in at least five individuals per continent, STRUCTURE modeling was able to detect coarse genetic structure in the native range. Estimates of support for population number (ln Pr[X|K]) peaked at K = 2, and these two subpopulations were partitioned geographically, with one region including individuals from Hungary and Romania in eastern Europe and the other region including individuals from Turkey and the Republic of Georgia in the Caucasus (Figure 6A). Including invading and naturalized individuals together with natives in a single model obscured all genetic structure and supported no subpopulations, even when geographic groups were provided as prior information in the model, a pattern indicative of a large number of admixed individuals in the dataset (Pritchard *et al.* 2000). When the two native subpopulations were instead provided as *a priori* fixed groups, invading and naturalized individuals were both assigned as admixtures of the native subpopulations (Figure 6B). The evidence for admixture suggested by our coarse population sampling is consistent with a history of multiple introductions, and similar patterns in the Spanish collections provide some of the first support for the hypothesis that western European populations are not native and are themselves naturalized products of past introductions (Maddox *et al.* 1985).

Inferences regarding introduction scenarios are only as robust as the sampling of the potential source populations (Dlugosch and Parker 2008), however, and our dataset is not extensive enough to rule out that unsampled source populations—rather than admixture of our observed native populations—have produced the current invasions. Thorough geographic sampling of the native range is essential for correctly identifying the most likely source genotypes. Moreover, dozens of individuals should be sampled from within each population to accurately estimate allele frequencies, and recover well-supported patterns of population structure (Pritchard *et al.* 2000; Avise 2004). Transcriptome studies generally have been considered too expensive to be used for this kind of broad population sampling, but as they becomes increasingly cost effective, investment in deeper sampling of individuals promises to be fruitful for robust population genomic studies.

### Conclusions

By analyzing the realized outcome of a wide range of sequencing efforts, our dataset reveals that allele recovery is far more dependent on sequencing depth than is gene recovery, although both are enhanced by additional sequencing depth. These relationships are of particular concern for diversity comparisons among outbred individuals, which are increasingly taking center stage as genomic sequencing moves outside of its historical focus on inbred lines of traditional model organisms. Modest transcriptome sampling nevertheless generates thousands of informative markers observable across many individuals. This is promising news for further studies of coding variation among individuals, and our ability to combine datasets generated with different methods over time – one of the inherent strengths of sequence-based population genetics. Using our novel bioinformatic pipeline for allele identification, we were able to recover previously hypothesized population features using 40 individuals dispersed across a worldwide distribution, demonstrating the information-rich nature of transcriptome populations datasets.

## Supplementary Material

Supporting Information
