# Supplementary material for: Allele Identification for Transcriptome-Based Population Genomics in the Invasive Plant Centaurea solstitialis
Source: G3 (Bethesda). 2013 Feb 1;3(2):359–67. doi: 10.1534/g3.112.003871 (PMC3564996; doi:10.1534/g3.112.003871)
Supplement: Supporting Information [file supp_3_2_359__index.html]

Supporting Information 

# Allele Identification for Transcriptome-Based Population Genomics in the Invasive Plant *Centaurea solstitialis*

## Supporting Information for Dlugosch *et al.*, 2013

**Files in this Data Supplement:**

- Supporting Information - Figures S1-S5 (PDF, 306 KB)
- Figure S1 - Contig numbers in transcriptome libraries sequenced with GS FLX Titanium (triangles) and GS FLX (circles) sequencing chemistry, as a function of total sequence effort after cleaning by SnoWhite (PDF, 78 KB)
- Figure S2 - Histograms of synonymous divergence at gene family nodes within five example C. *solstitialis transcriptomes*: invaders CA-4-4 and AR-8-15, naturalized SP-2-2, and native RO-1-6 and TK-1-5 (PDF, 129 KB)
- Figure S3 - Histogram of the number of sequence clusters (putative loci) aligning to a single Ultra Conserved Ortholog (UCO) (PDF, 306 KB)
- Figure S4 - GO Slim annotation categories for all clusters (dark bars) and inferred single loci (open bars) within the (A) Biological Processes, (B) Cellular Components, and (C) Molecular Function categories (PDF, 146 KB)
- Figure S5 - Histogram of the number of individual libraries covering the same SNP locus, among the top ten libraries (PDF, 73 KB)
