# Supplementary material for: Allele Identification for Transcriptome-Based Population Genomics in the Invasive Plant Centaurea solstitialis
Source: G3 (Bethesda). 2013 Feb 1;3(2):359–67. doi: 10.1534/g3.112.003871 (PMC3564996; doi:10.1534/g3.112.003871)
Supplement: Supporting Information [file supp_3.2.359_003871SI.pdf]

G3·Genes | Genomes | Genetics

Allele identification for transcriptome-based population genomics in the invasive plant *Centaurea solstitialis*

Katrina M. Dlugosch[*,†,1,2], Zhao Lai[‡,2], Aurélie Bonin[‡], José Hierro[**], and Loren H. Rieseberg[*,‡]

[*] Department of Botany, University of British Columbia, Vancouver, BC V6T1Z4 Canada
[†] Department of Ecology and Evolutionary Biology, University of Arizona, Tucson, AZ 85721
[‡] Department of Biology and Center for Genomics and Bioinformatics, Indiana University, Bloomington, IN 47405
[**] Facultad de Ciencias Exactas y Naturales, INCITAP (CONICET-Universidad Nacional de La Pampa), AR-6300, Santa Rosa, La Pampa, Argentina

[1] Author for correspondence: kdlugosch@email.arizona.edu
[2] These authors contributed equally to this work.

Publicly available data:
NCBI accession number #SRA059334

DOI: 10.1534/g3.112.003871

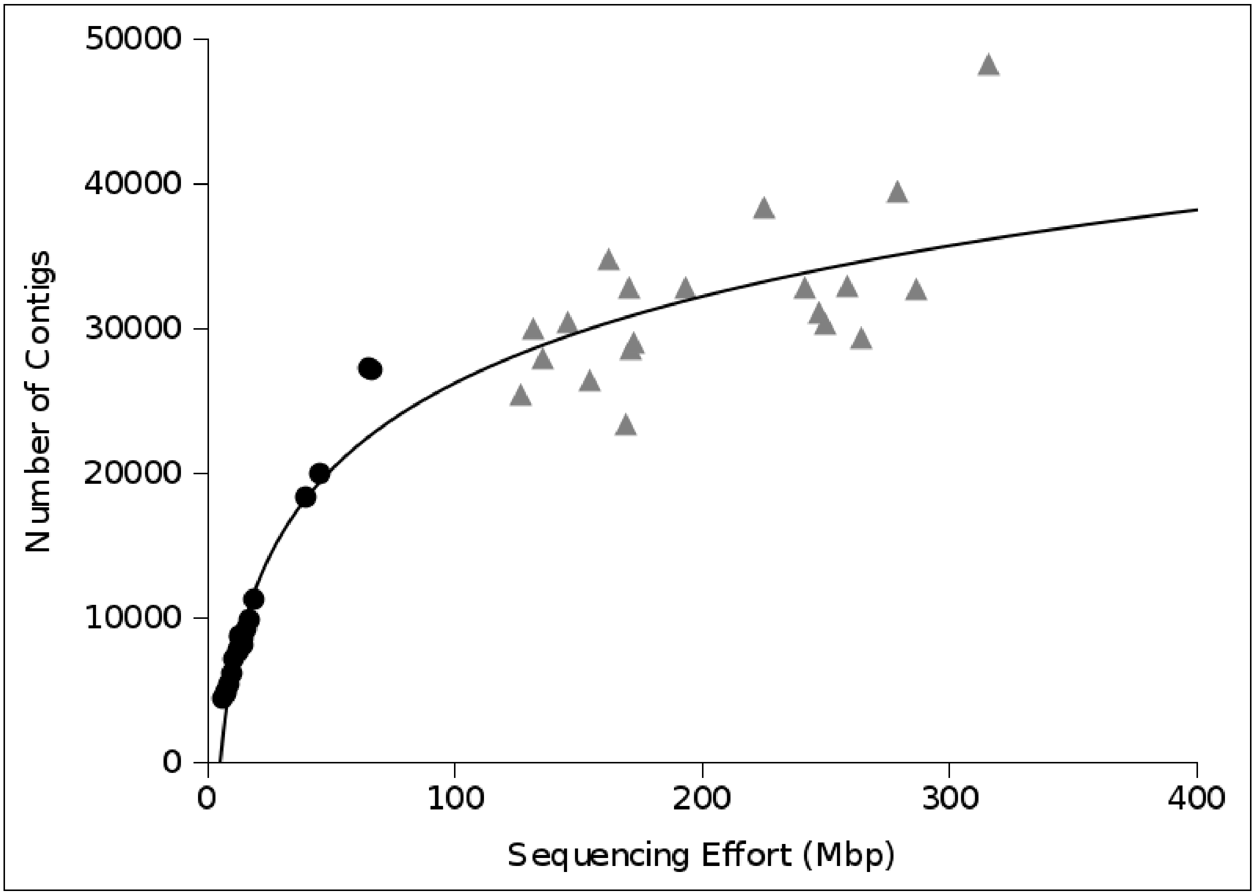

Figure S1 Contig numbers in transcriptome libraries sequenced with GS FLX Titanium (triangles) and GS FLX (circles) sequencing chemistry, as a function of total sequence effort after cleaning by SnoWhite. A logarithmic fit is shown for variation across all individuals.

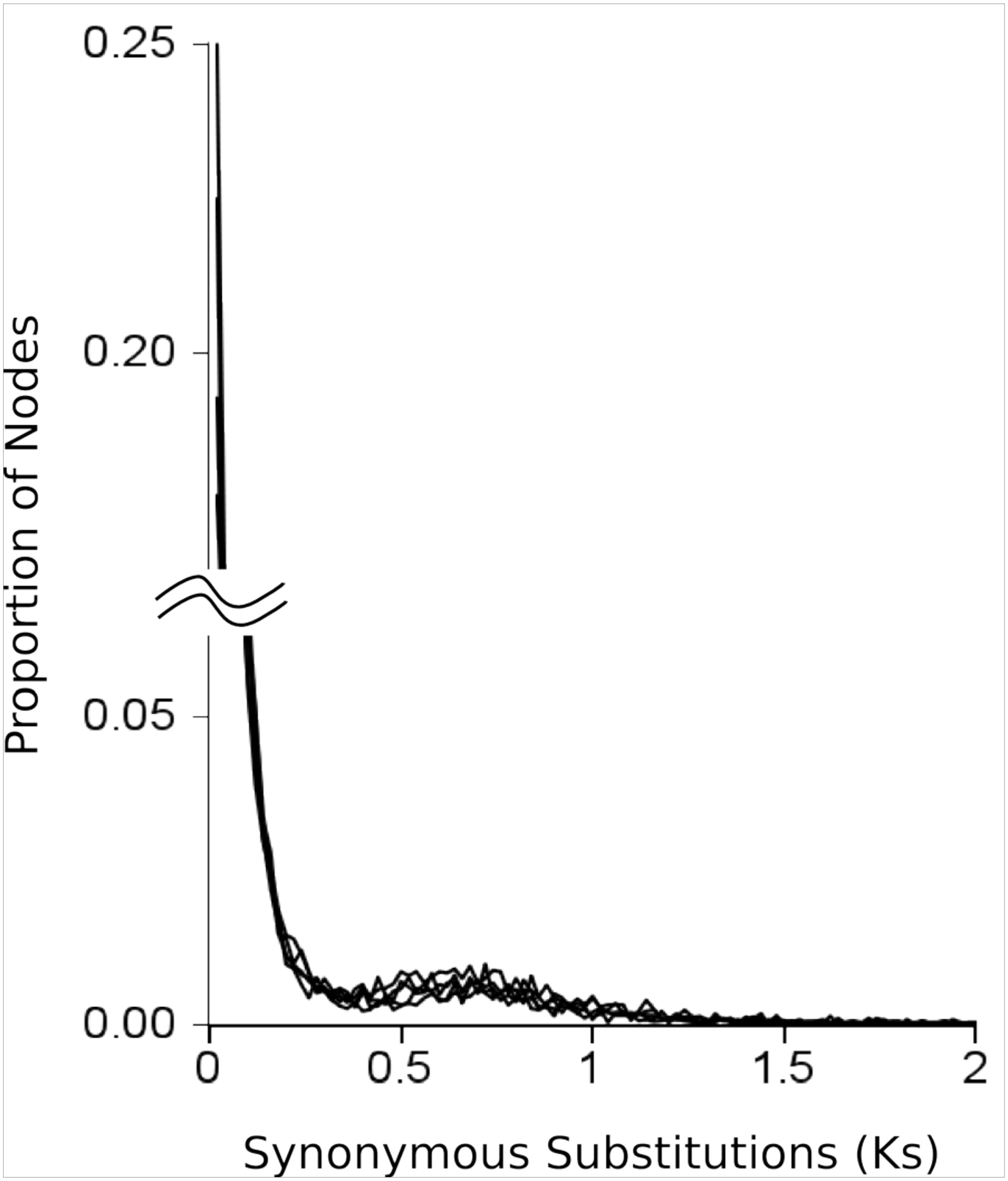

Figure S2 Histograms of synonymous divergence at gene family nodes within five example C*. solstitialis* transcriptomes: invaders CA-4-4 and AR-8-15, naturalized SP-2-2, and native RO-1-6 and TK-1-5. The small peak centered at ~0.65 Ks corresponds to an ancient genome duplication event near the base of the Compositae (Barker et al. 2008).

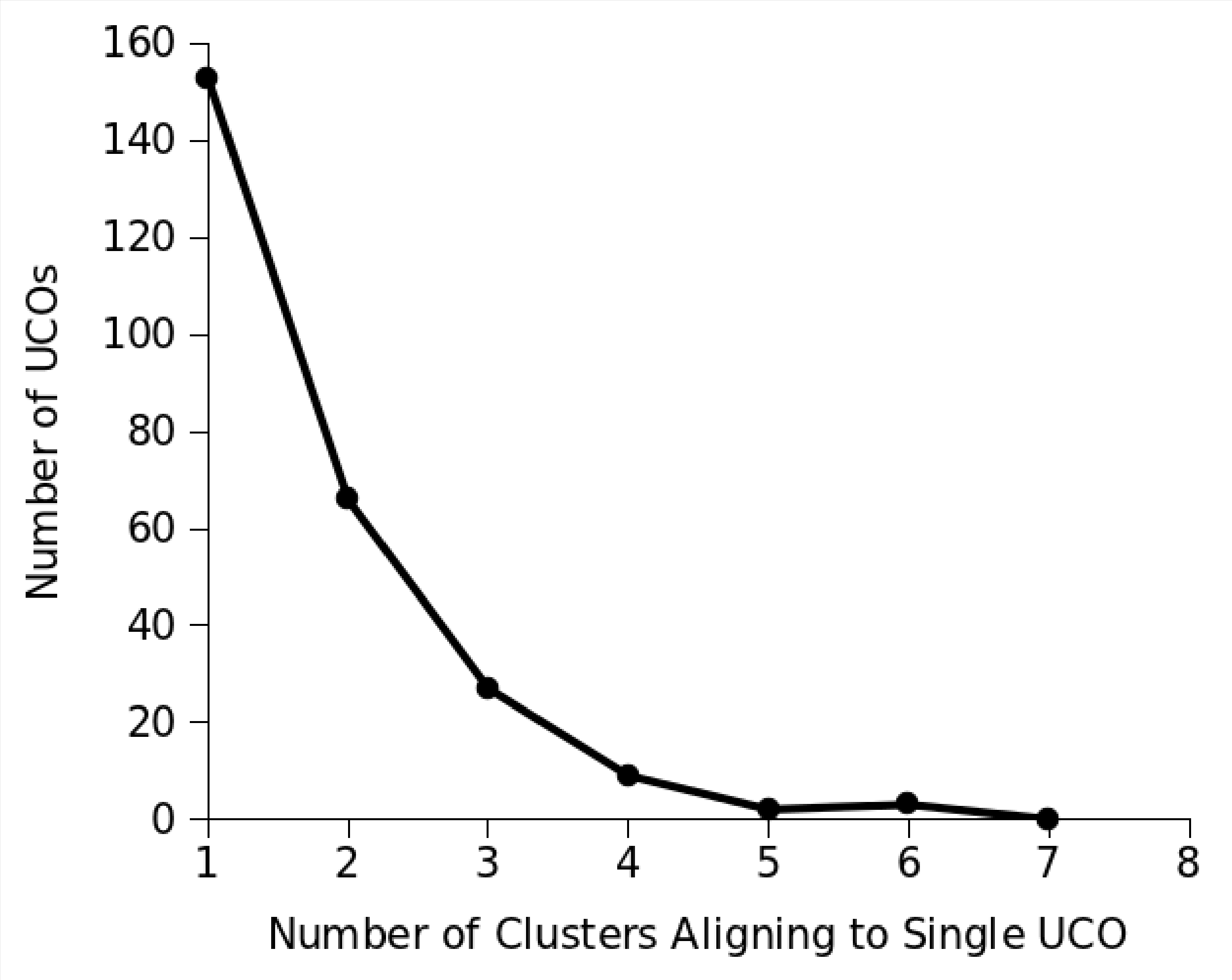

Figure S3 Histogram of the number of sequence clusters (putative loci) aligning to a single Ultra Conserved Ortholog (UCO). UCOs are expected to be single copy. One-to-one matches of a cluster and a UCO are expected for accurately clustered alleles.

A

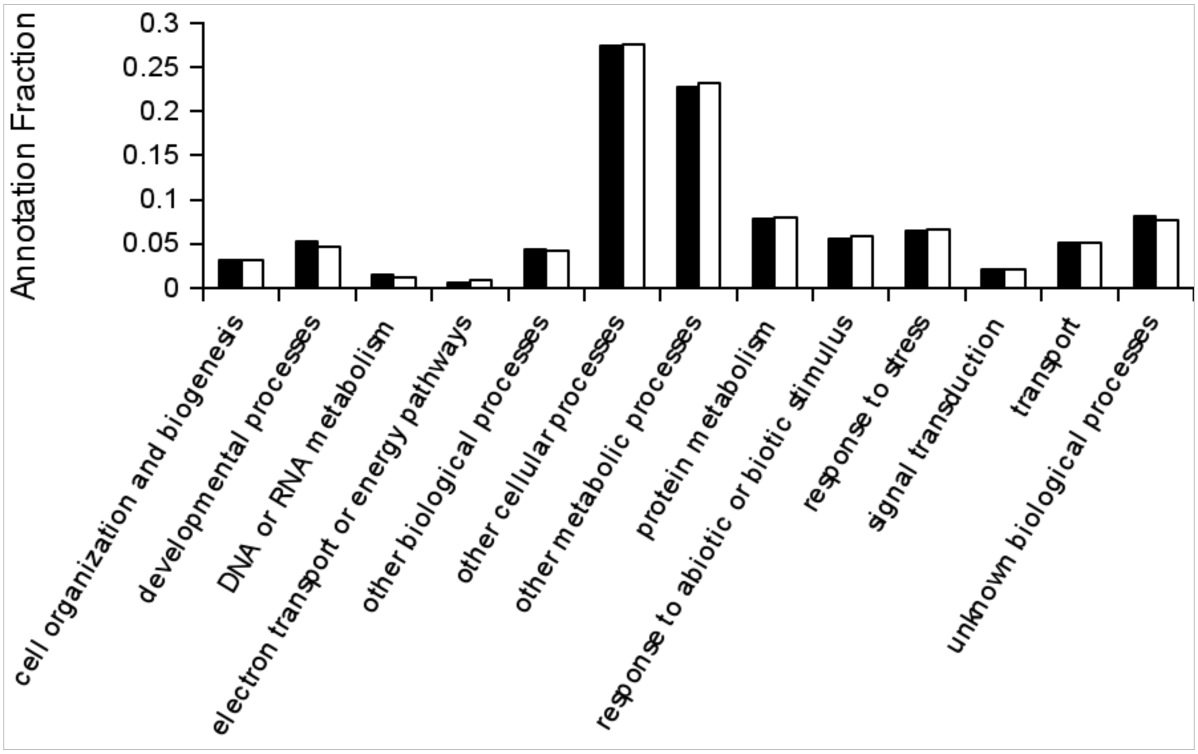
Annotation Fraction
0.3
0.25
0.2
0.15
0.1
0.05
0
cell organization and biogenesis
developmental processes
DNA or RNA metabolism
electron transport or energy pathways
other biological processes
other cellular processes
other metabolic processes
protein metabolism
response to abiotic or biotic stimulus
response to stress
signal transduction
transport
unknown biological processes

B

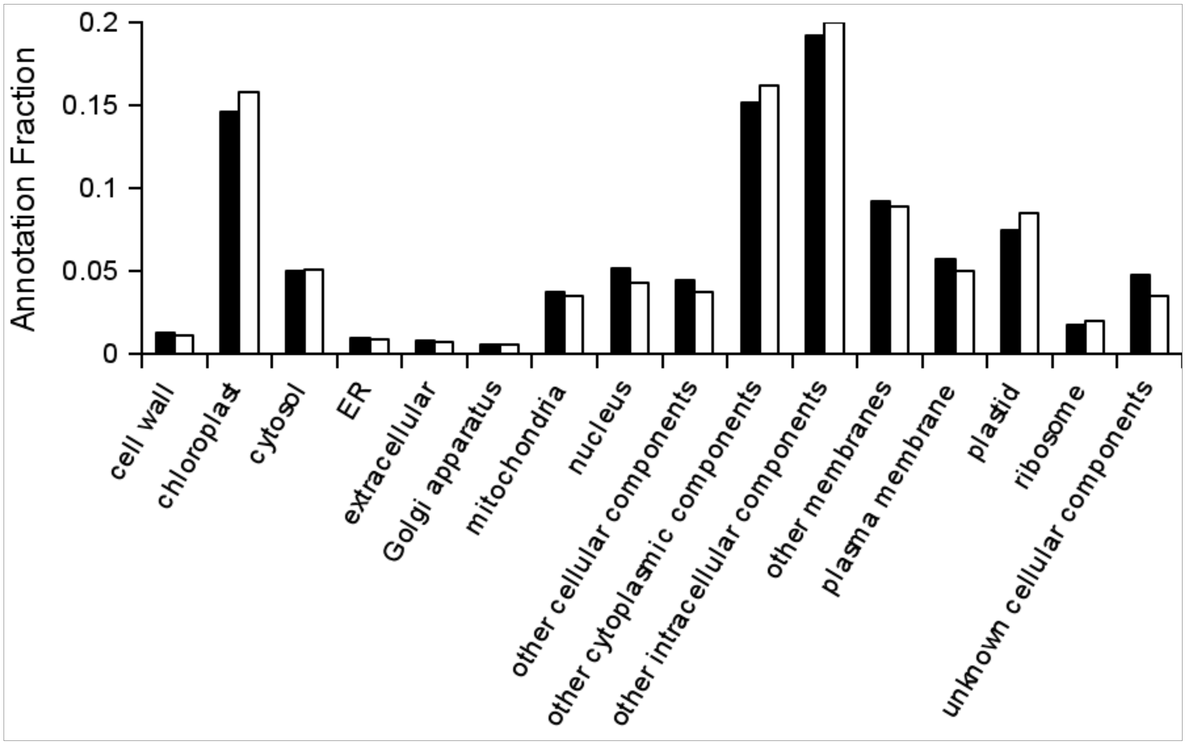
Annotation Fraction
0.2
0.15
0.1
0.05
0
cell wall
chloroplast
cytosol
ER
extracellular
Golgi apparatus
mitochondria
nucleus
other cellular components
other cytoplasmic components
other intracellular components
other membranes
plasma membrane
plastid
ribosome
unknown cellular components

C

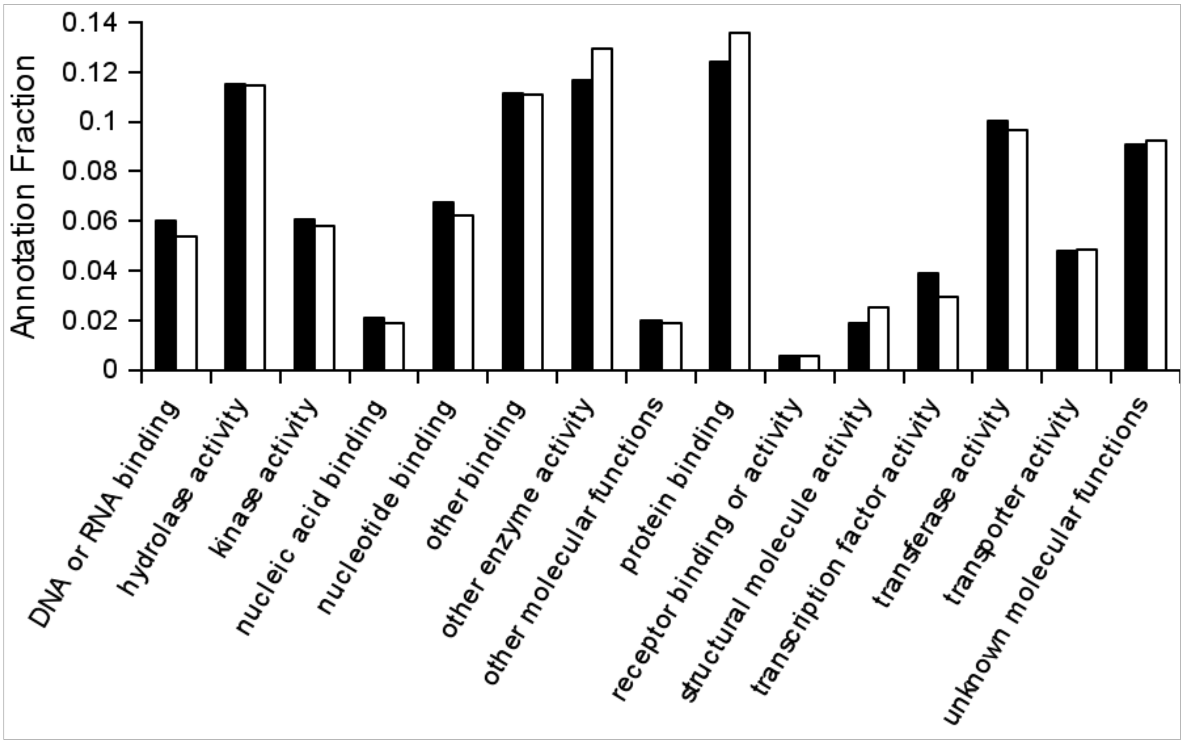

Figure S4 GO Slim annotation categories for all clusters (dark bars) and inferred single loci (open bars) within the (A) Biological Processes, (B) Cellular Components, and (C) Molecular Function categories.

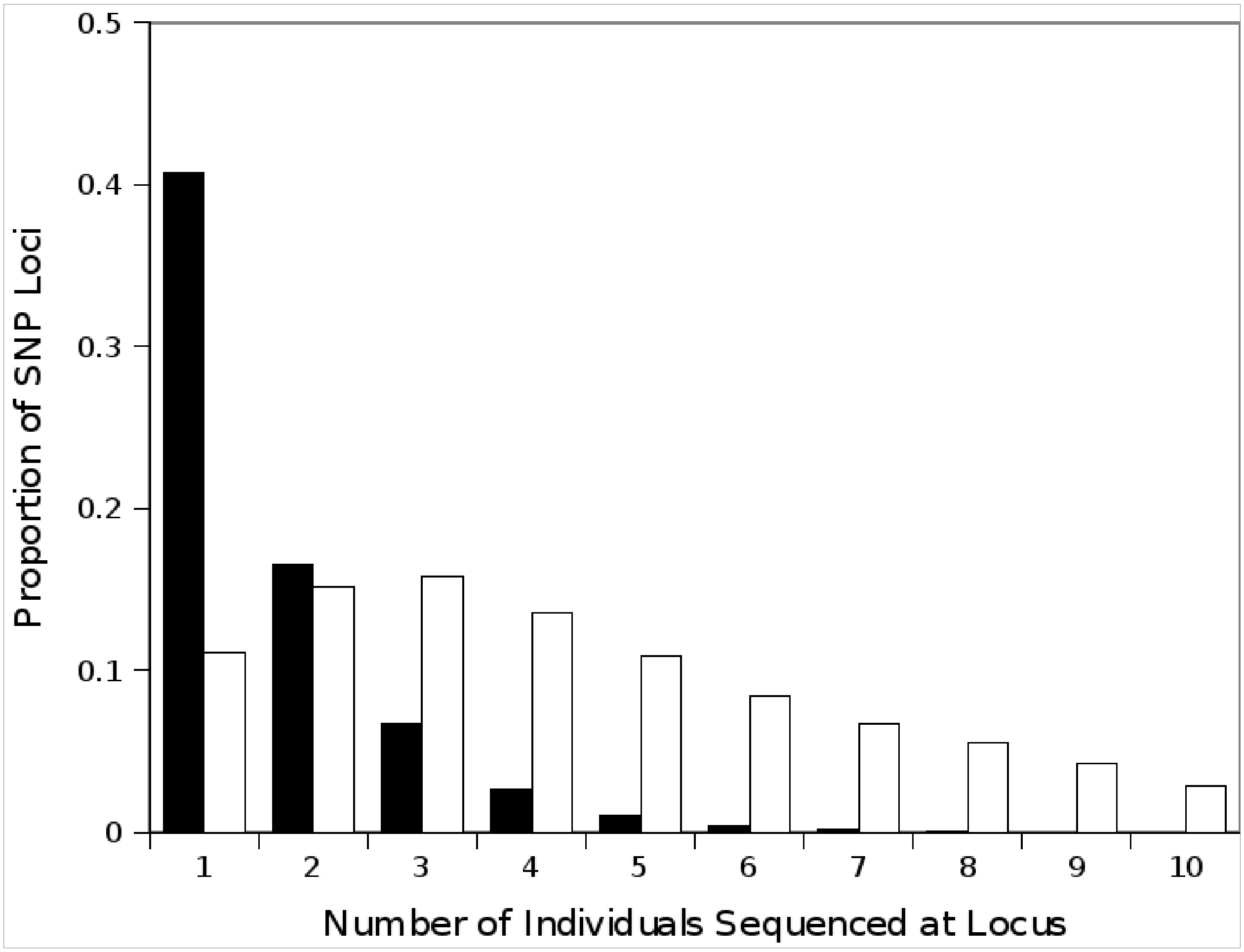

Figure S5 Histogram of the number of individual libraries covering the same SNP locus, among the top ten libraries. All ten libraries are derived from >200 Mb sequencing effort. Observed values (open bars) show significantly greater sharing of SNP positions than expected by chance (black bars).
