# Supplementary material for: Allele Identification for Transcriptome-Based Population Genomics in the Invasive Plant Centaurea solstitialis
Source: G3 (Bethesda). 2013 Feb 1;3(2):359–67. doi: 10.1534/g3.112.003871 (PMC3564996; doi:10.1534/g3.112.003871)
Supplement: Supporting Information [file supp_3.2.359_FigureS1.pdf]

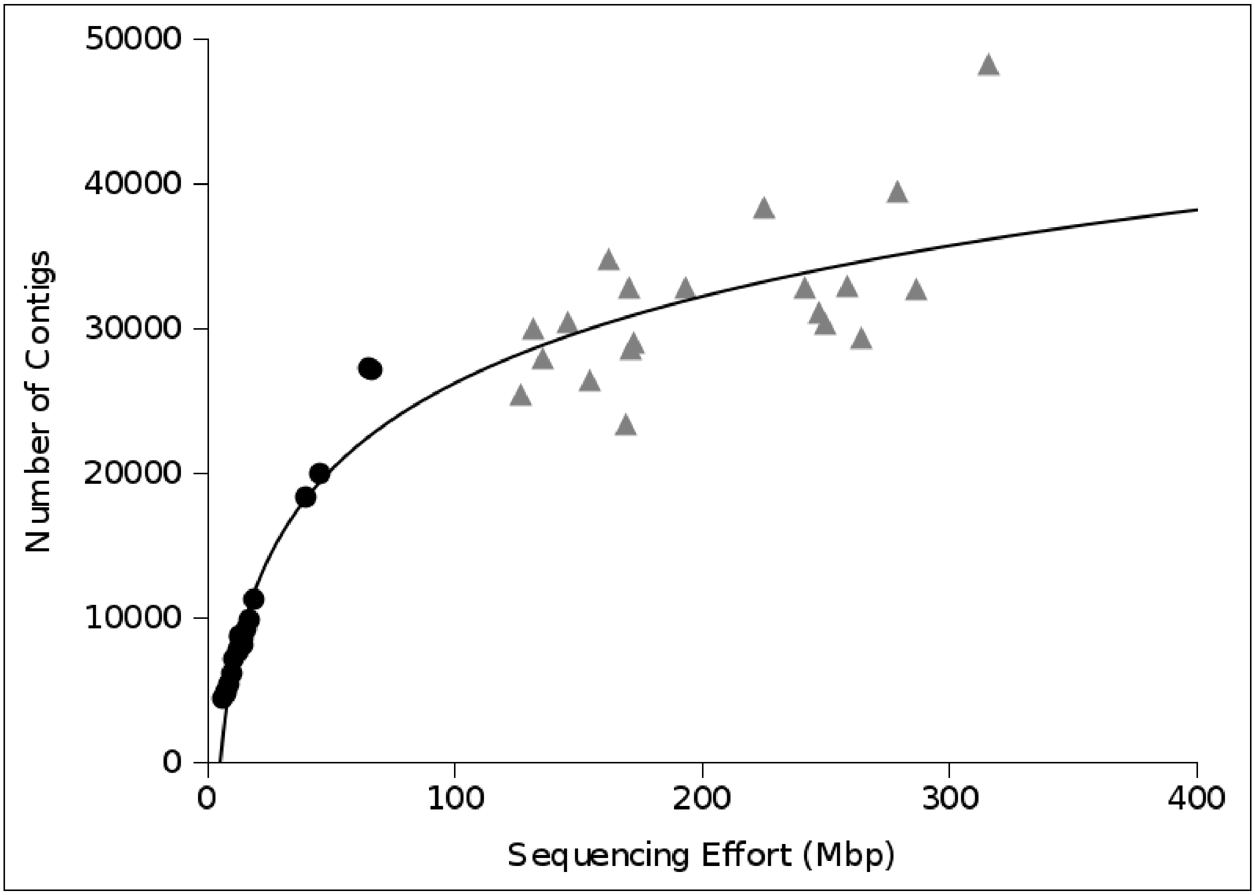

Figure S1 Contig numbers in transcriptome libraries sequenced with GS FLX Titanium (triangles) and GS FLX (circles) sequencing chemistry, as a function of total sequence effort after cleaning by SnoWhite. A logarithmic fit is shown for variation across all individuals.
