# Supplementary material for: Allele Identification for Transcriptome-Based Population Genomics in the Invasive Plant Centaurea solstitialis
Source: G3 (Bethesda). 2013 Feb 1;3(2):359–67. doi: 10.1534/g3.112.003871 (PMC3564996; doi:10.1534/g3.112.003871)
Supplement: Supporting Information [file supp_3.2.359_FigureS2.pdf]

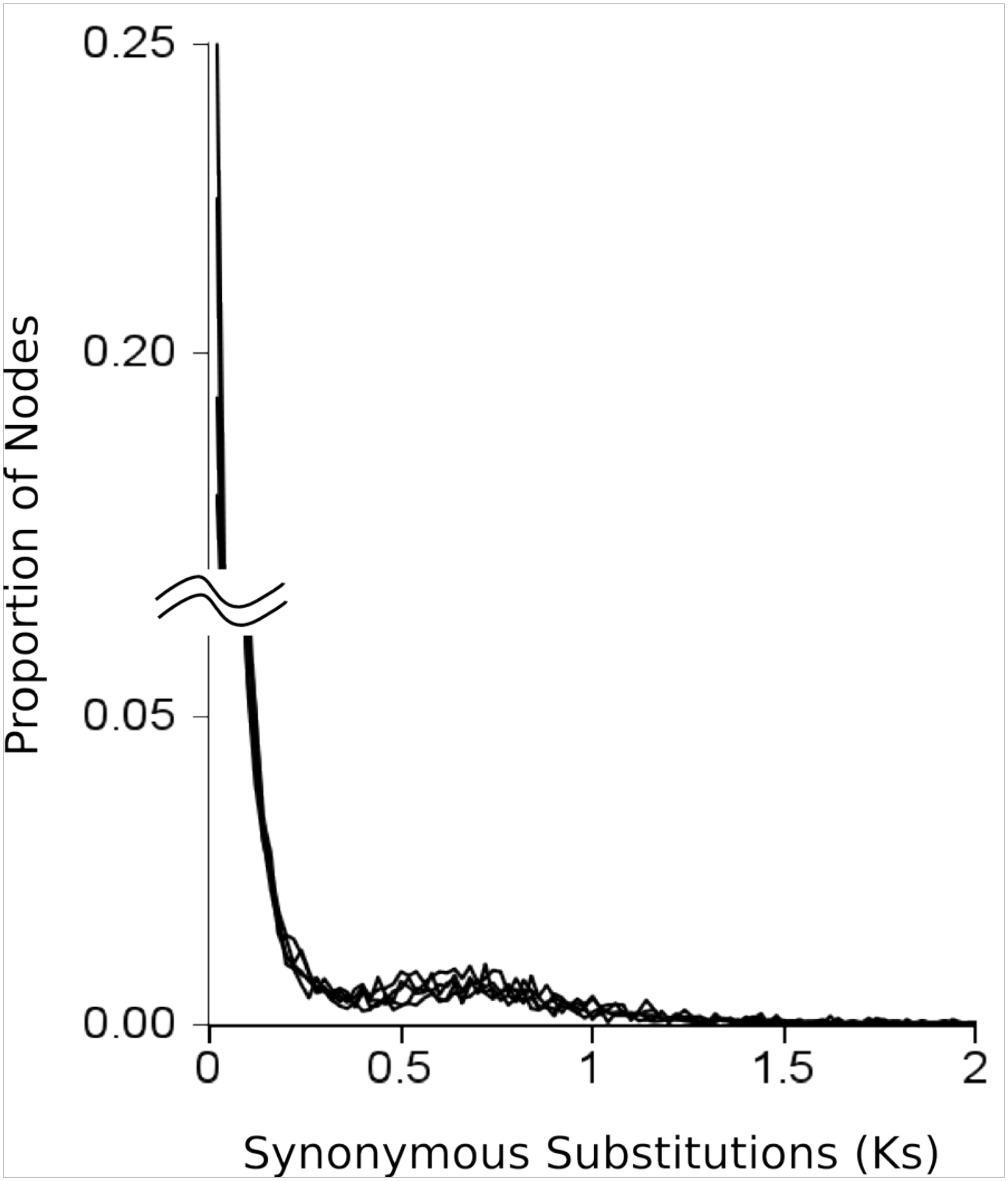

Figure S2 Histograms of synonymous divergence at gene family nodes within five example C*. solstitialis* transcriptomes: invaders CA-4-4 and AR-8-15, naturalized SP-2-2, and native RO-1-6 and TK-1-5. The small peak centered at ~0.65 Ks corresponds to an ancient genome duplication event near the base of the Compositae (Barker et al. 2008).
