# Supplementary material for: Allele Identification for Transcriptome-Based Population Genomics in the Invasive Plant Centaurea solstitialis
Source: G3 (Bethesda). 2013 Feb 1;3(2):359–67. doi: 10.1534/g3.112.003871 (PMC3564996; doi:10.1534/g3.112.003871)
Supplement: Supporting Information [file supp_3.2.359_FigureS3.pdf]

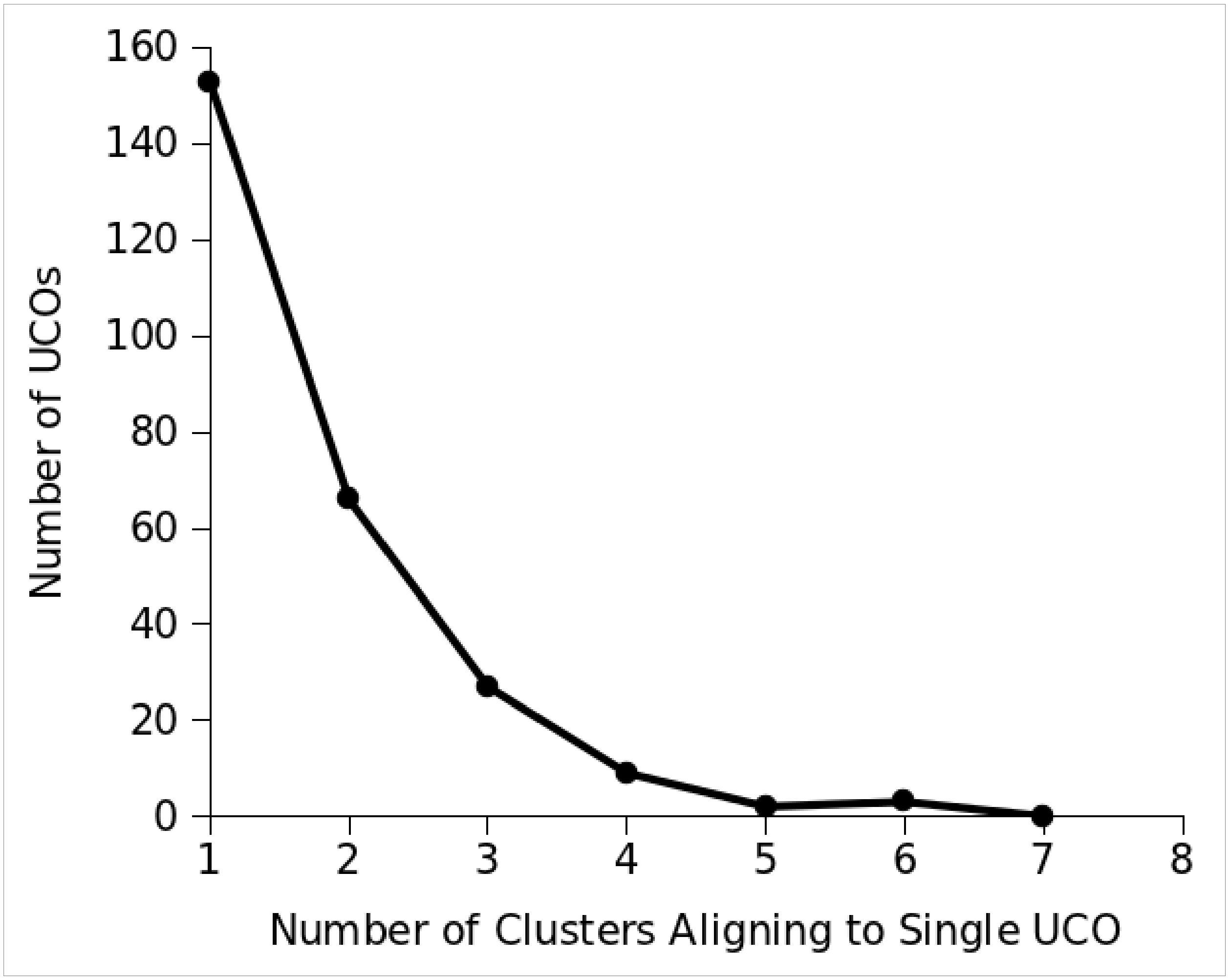

Figure S3 Histogram of the number of sequence clusters (putative loci) aligning to a single Ultra Conserved Ortholog (UCO). UCOs are expected to be single copy. One-to-one matches of a cluster and a UCO are expected for accurately clustered alleles.
