# Supplementary material for: Allele Identification for Transcriptome-Based Population Genomics in the Invasive Plant Centaurea solstitialis
Source: G3 (Bethesda). 2013 Feb 1;3(2):359–67. doi: 10.1534/g3.112.003871 (PMC3564996; doi:10.1534/g3.112.003871)
Supplement: Supporting Information [file supp_3.2.359_FigureS4.pdf]

A

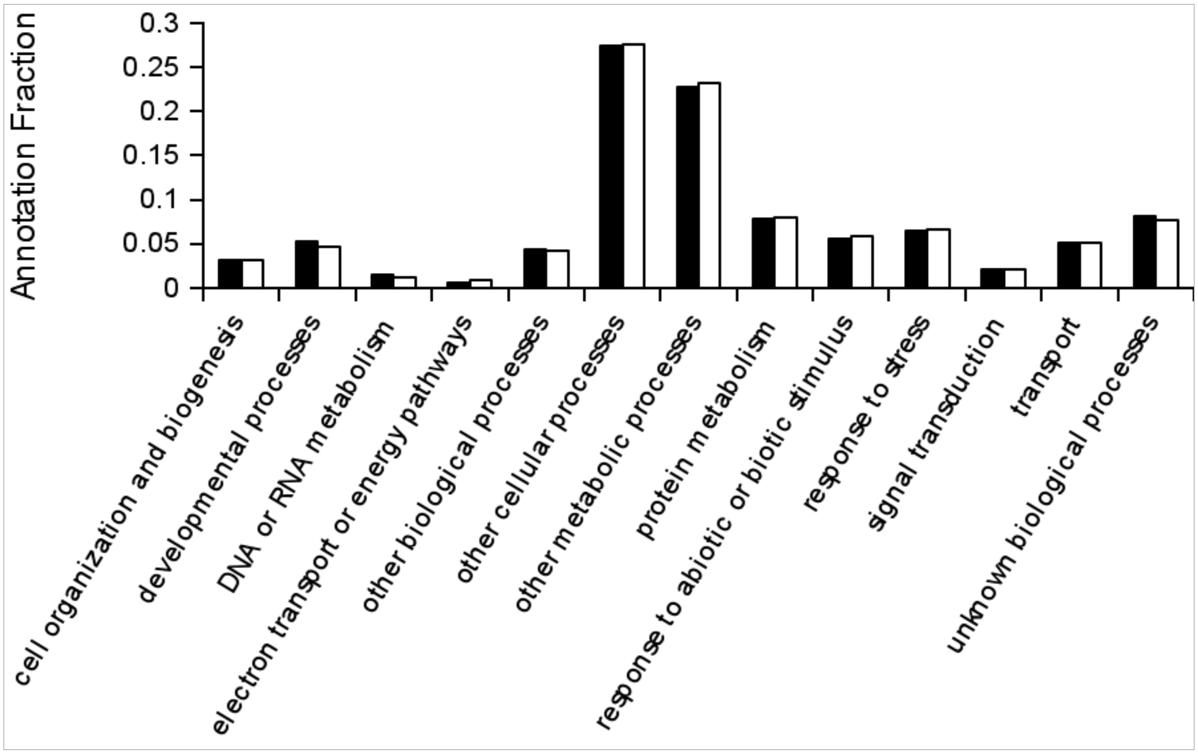
Annotation Fraction
0.3
0.25
0.2
0.15
0.1
0.05
0
cell organization and biogenesis
developmental processes
DNA or RNA metabolism
electron transport or energy pathways
other biological processes
other cellular processes
other metabolic processes
protein metabolism
response to abiotic or biotic stimulus
response to stress
signal transduction
transport
unknown biological processes

B

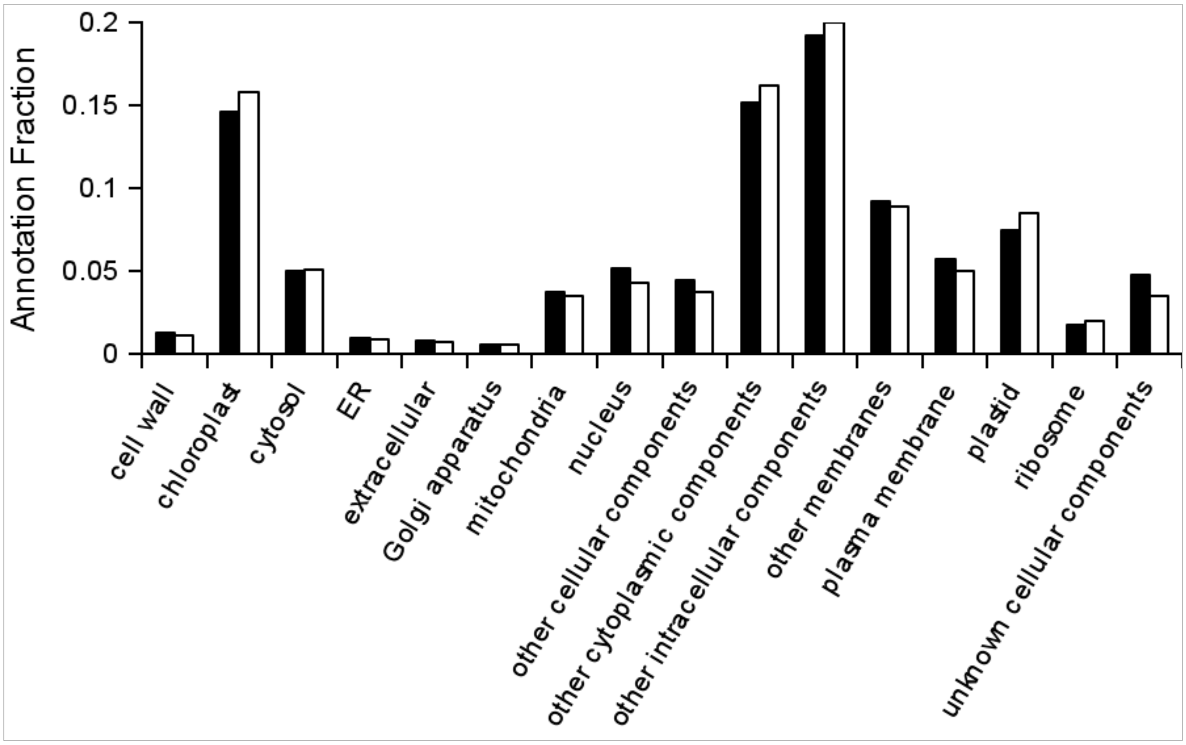
Annotation Fraction
0.2
0.15
0.1
0.05
0
cell wall
chloroplast
cytosol
ER
extracellular
Golgi apparatus
mitochondria
nucleus
other cellular components
other cytoplasmic components
other intracellular components
other membranes
plasma membrane
plastid
ribosome
unknown cellular components

C

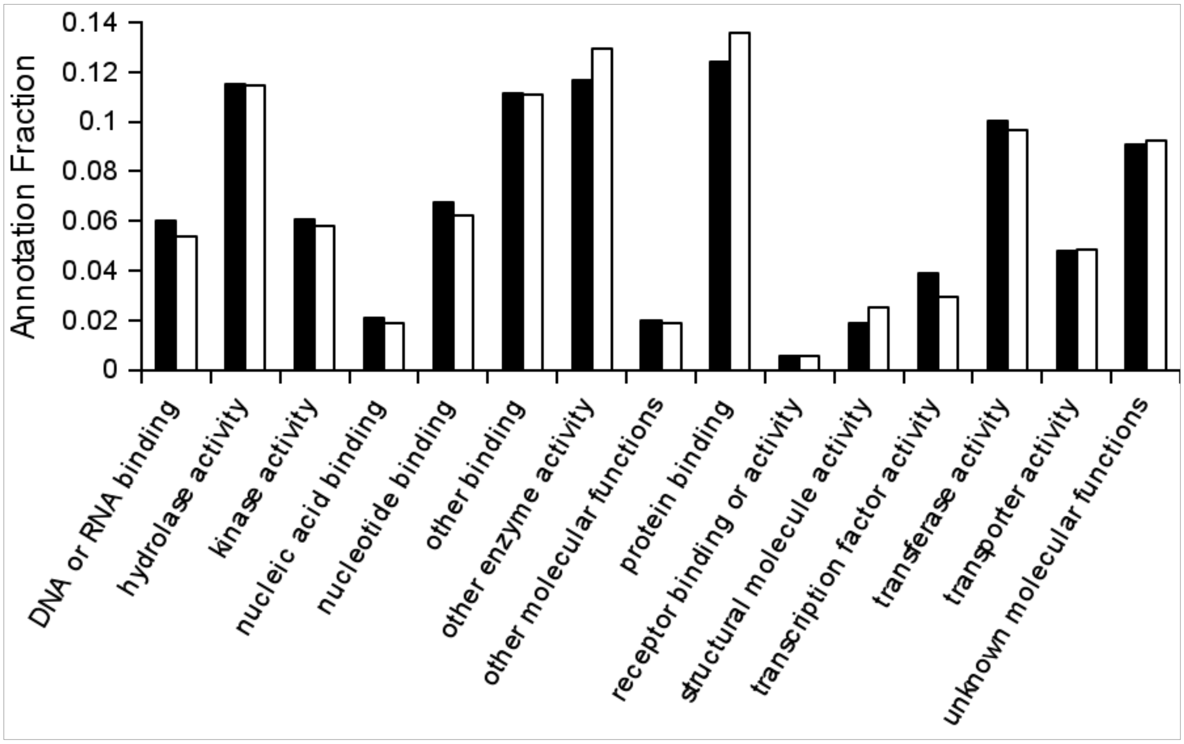

Figure S4 GO Slim annotation categories for all clusters (dark bars) and inferred single loci (open bars) within the (A) Biological Processes, (B) Cellular Components, and (C) Molecular Function categories.
