# Supplementary material for: Allele Identification for Transcriptome-Based Population Genomics in the Invasive Plant Centaurea solstitialis
Source: G3 (Bethesda). 2013 Feb 1;3(2):359–67. doi: 10.1534/g3.112.003871 (PMC3564996; doi:10.1534/g3.112.003871)
Supplement: Supporting Information [file supp_3.2.359_FigureS5.pdf]

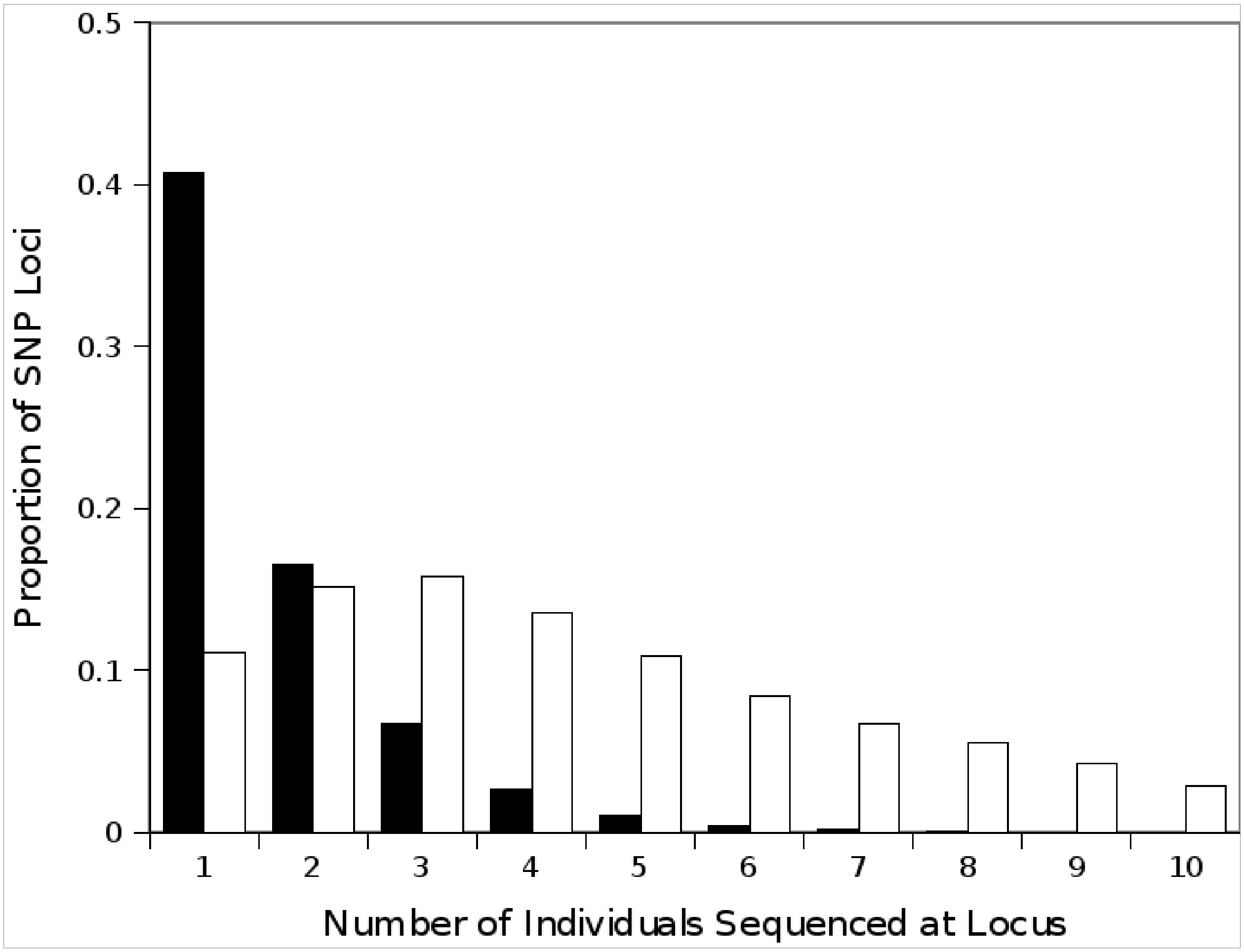

Figure S5 Histogram of the number of individual libraries covering the same SNP locus, among the top ten libraries. All ten libraries are derived from >200 Mb sequencing effort. Observed values (open bars) show significantly greater sharing of SNP positions than expected by chance (black bars).
